# Susceptibility of Human Placenta Derived Mesenchymal Stromal/Stem Cells to Human Herpesviruses Infection

**DOI:** 10.1371/journal.pone.0071412

**Published:** 2013-08-05

**Authors:** Simone Avanzi, Valerio Leoni, Antonella Rotola, Francesco Alviano, Liliana Solimando, Giacomo Lanzoni, Laura Bonsi, Dario Di Luca, Cosetta Marchionni, Gualtiero Alvisi, Alessandro Ripalti

**Affiliations:** 1 Department of Oncology, Haematology and Laboratory Medicine, Operative Unit of Microbiology, A. O-U. di Bologna Policlinico S. Orsola-Malpighi, Bologna, Italy; 2 Department of Haematology and Oncological Sciences “L.A. Seragnoli”, Microbiology Section, University of Bologna, Bologna, Italy; 3 Department of Experimental and Diagnostic Medicine, Microbiology Section, University of Ferrara, Ferrara, Italy; 4 Department of Histology, Embryology and Applied Biology, University of Bologna, Bologna, Italy; 5 Department of Molecular Medicine, Microbiology Section University of Padua, Padua, Italy; Shanghai Jiao Tong University School of Medicine, China

## Abstract

Fetal membranes (FM) derived mesenchymal stromal/stem cells (MSCs) are higher in number, expansion and differentiation abilities compared with those obtained from adult tissues, including bone marrow. Upon systemic administration, *ex vivo* expanded FM-MSCs preferentially home to damaged tissues promoting regenerative processes through their unique biological properties. These characteristics together with their immune-privileged nature and immune suppressive activity, a low infection rate and young age of placenta compared to other sources of SCs make FM-MSCs an attractive target for cell-based therapy and a valuable tool in regenerative medicine, currently being evaluated in clinical trials. In the present study we investigated the permissivity of FM-MSCs to all members of the human *Herpesviridae* family, an issue which is relevant to their purification, propagation, conservation and therapeutic use, as well as to their potential role in the vertical transmission of viral agents to the fetus and to their potential viral vector-mediated genetic modification. We present here evidence that FM-MSCs are fully permissive to infection with Herpes simplex virus 1 and 2 (HSV-1 and HSV-2), Varicella zoster virus (VZV), and Human Cytomegalovirus (HCMV), but not with Epstein-Barr virus (EBV), Human Herpesvirus-6, 7 and 8 (HHV-6, 7, 8) although these viruses are capable of entering FM-MSCs and transient, limited viral gene expression occurs. Our findings therefore strongly suggest that FM-MSCs should be screened for the presence of herpesviruses before xenotransplantation. In addition, they suggest that herpesviruses may be indicated as viral vectors for gene expression in MSCs both in gene therapy applications and in the selective induction of differentiation.

## Introduction

Nonembryonic stem cells (SCs) opened new avenues in developmental biology and regenerative medicine. Mesenchymal stromal/cells (MSCs) [1] constitute a heterogeneous population found first in bone marrow (BM) [2]. MSCs are easy to isolate [3], they have a superior expansion potential as compared to other adult tissue-derived SCs, and are endowed with low inherent immunogenicity and the ability of modulating/suppressing immunologic responses [4]. These characteristics together with high plasticity, a tendency to migrate into damaged tissues where they orchestrate regenerative processes, and their outstanding record of safety in clinical trials make these cells prime candidates for cellular therapy. Indeed MSCs from BM or umbilical cord blood have been used in therapeutic approaches involving hematopoietic, cardiovascular, central nervous, gastrointestinal, renal, and orthopedic systems, as well as in the temptative treatment of genetic disorders and cancer [4], [5], and are being considered for gene therapy [6], [7].

Adult BM is the common source of MSCs for clinical use [5], however the frequency of MSCs in human adult BM is relatively low, and availability is conditional to invasive procedures. As a consequence a quest for alternative sources of MSCs was initiated, resulting in finding MSCs in multiple adult and neonatal tissues like fat, skin, cartilage, skeletal muscle, synovium, peripheral blood, dental pulp, umbilical cord, amniotic fluid and placenta [3], [8]–[10]. The human placenta at term is an alternative, ethically acceptable, and easily available source of MSCs. Importantly, a single amnion membrane can yield between 1–4×10^7^ stromal cells, approximately half of what is expected from a term chorion membrane. This large yield of cells allows for obtaining suitable amounts of FM-MSCs for cell therapy upon a limited number of passages, and warrants maximal preservation of the phenotypical characteristics of the original population of cells. Furthermore fetal membranes (FM) derived-MSCs are characterized by high plasticity [11]–[13], and are capable of differentiating into both their natural mesodermal and non mesodermal lineages [14]–[16], suggesting similar characteristics as BM-MSCs [17]. Amniotic membranes contribute to fetal maternal tolerance [18] and their allogenic transplantation, or transplantation of cells derived from them, does not induce acute immune rejection even in the absence of immunosuppression [19]–[21]. It is not surprising therefore that FM-MSCs do not elicit allogeneic or xenogeneic immune responses, and are able to actively suppress lymphocyte proliferation [22]–[24]. Accordingly FM-MSCs are considered a promising source of cells with clinical applications in allogenic transplantation, as in heterologous peripheric revascularization, and are being evaluated for their immunomodulatory properties [25]–[29]. In addition to the above mentioned therapeutic applications of MSCs, FM-MSCs are expected to be clinically used as autologous grafts for fetuses and newborns in *peripartum* tissue regeneration or for *in utero* transplantation in case of genetic disorders without immunologic rejection by the recipient [30]–[32], proof of principle having already been established [33], [34]. Lastly, gene transfer in fetal blood derived MSCs with unperturbed differentiation potential has been performed [35] and the possible use of FM-MSCs in antitumor therapeutic strategies has been confirmed [36], [37], paving the way to their potential use in gene therapy approaches, and large scale production and manufacturing for clinical trials is being implemented [38]–[40].

Infections by herpesviruses are a common complication in the transplant and pregnancy settings. The human *Herpesviridae* family is composed of large, enveloped DNA viruses with close structural similarity and includes the Herpes simplex viruses types 1 and 2 (HSV-1 and 2), Varicella zoster virus (VZV), Epstein Barr virus (EBV), Human Cytomegalovirus (HCMV), as well as Human Herpesvirus (HHV) types 6, 7 and 8. All members of the family replicate in the nucleus of the infected cell after activating a coordinated cascade of mRNA synthesis, that allows the distinction of gene transcription into three temporal classes: immediate early (IE), early (E) and late (L). These viruses all share the ability to establish latency and reactivate at a later time, and all are human pathogens. Allogeneic SC transplantation is often complicated by reactivation of herpesviruses, which are also a cause of morbidity and mortality in the solid organ transplant setting [41]. The presence of selected viruses in MSCs from donor BM has been investigated [42]–[44], human fetal BM-MSC support transcription of HHV-8 LANA/ORF73 messenger (ORF73 from now on) [44], while adult BM-MSCs have been proved permissive to HSV-1 and HCMV [43], [45], rising concerns regarding their possible transmission to transplant recipients. Furthermore several herpesviruses can establish congenital infections: structural fetal abnormalities can result from intrauterine infection and transmission of the infection during pregnancy or at the time of delivery and can result in severe neonatal disease [46].

In the present study we investigated the susceptibility of FM-MSCs to all members of the human *Herpesviridae* family. As a whole, studying FM-MSCs permissivity to herpesviruses is relevant to: (i) the transplantation field, in that infection of plastic cells used in regenerative medicine pre- or post-transplant could interfere with their differentiation process and compromise the engraftment, inducing cytopathology or death of the implanted cells; (ii) vertical transmission of infections, a setting where mechanisms of virus spread through the placenta are vastly unexplored, and the consequences of congenital infection have been poorly characterized for most members of the family, especially for what pertains to stillbirth and post-partum long term sequelae; (iii) gene therapy and viral vector mediated genetic modification of SCs.

Our results clearly show that FM-MSCs are fully permissive to infection by HSV-1/2, VZV and CMV. On the other hand, despite EBV, HHV-6, HHV-7 and HHV-8 being capable of entering into FM-MSCs, only limited gene expression occurs, resulting in a nonproductive infection.

## Materials And Methods

### Antibodies

#### Surface antigens analysis

Mouse monoclonal antibodies anti-CD21 (clone LB21) and anti-CD35 (clone E11), were purchased from Antibodies-Online (Antibodies-Online INC., Atlanta, USA); anti-CD19 (clone HD37) was obtained from DAKO (DAKO, Glostrup, Denmark); anti-CD45 (clone 2D1, APC), CD11c (S-HCL-3, PE), CD14 (MΦP9, PE), CD31 (WM-59, PE), CD36 (NL07, FITC), CD90 (5E10, FITC), CD59 (P282-H19, PE), CD184 (12G5, PE) and CD166 (3A6, PE) were purchased form BD (Becton-Dickinson and Company, New York, USA); anti-CD106, (1G1b1-PE) was obtained from Southern Biotechnology (Southern Biotechnology Associates, Birmingham, USA); anti-HLA-DR (Tu36, FITC), CD105 (SN6, PE), CD44 (MEM 85, FITC), HLA-ABC (Tu149, FITC), CD80 (MEM-233, PE) and CD29 (MEM101A, PE) were purchased from Caltag Laboratories (Caltag Laboratories, Bangkok, Thailand), anti-CD31 (CBL468F, FITC) and CD34 (HPCA-2, Bdis, APC or PE-Cy7) were obtained from Cymbus Biotechnology (Cymbus Biotechnology, Chandlers Ford, UK).

#### Indirect ImmunoFluorescence (IIF)

Monoclonal mouse antibody raised against HSV-1 and HSV-2 gD, clone HDI was a kind gift from Gabriella Campadelli-Fiume (Bologna, Italy). HCMV anti-IE clone E13 and anti-pp65, clone 1C3 were purchased from ARGENE SA (Varilhes, France). Anti-UL44 clone 10D8 was obtained from Virusys (Virusys corp., Taneytown, USA). Fluorescein (clone sc2010) or Rodhamine (clone sc2092) conjugated secondary antibodies were purchased from Santa Cruz (Santa Cruz Biotechnology Inc., Santa Cruz, USA). All antibodies were diluted in PBS, according to manufacturer's indications.

### Cells

Peripheral blood mononuclear cells (PBMCs) were isolated by Ficoll-Hypaque density centrifugation of adult buffy coat samples (S.Orsola-Malpighi University Hospital, Bologna, Italy) and primary human B lymphocytes were positively selected by immunomagnetic cell isolation using CD19 Pan B Dynabeads (Life Technologies, Austin, USA) followed by bead detachment. Purified B-Cells were mainteined in RPMI 1640 medium supplemented with 10% FCS. Human embryo lung derived fibroblasts (HEL; ATCC CCL-137) were cultured in minimal essential medium (MEM, PAA, Pasching, China), supplemented with 10% fetal calf serum (FCS) and 100 U/ml penicillin and 100 µg/ml streptomycin (P/S). Human umbilical vascular endothelial cells (HUVECs; ATCC PCS-100-013), were maintained in endothelial cell growth medium supplemented with 5% FCS and growth factors (EGM-MV single aliquots, both from BioWhittaker, Cambrex Bio Science, Walkersville, MD). Vero (ATCC CCL-81) and baby hamster kidney (BHK; ATCC CCL-10) cells were maintained in Dulbecco's modified Eagle's medium (DMEM, EuroClone, Milan, Italy) supplemented with 5% FCS, and P/S. T lymphoid J-Jhan [47] and Sup T1 (ATCC CRL-1942) as well as B lymphoid B95-8 (ECACC 85011419) and BC-3 (ATCC CRL-2277) cell lines were grown in suspension in RPMI 1640 (EuroClone, Milan, Italy) supplemented with 10% FCS.

### FM-MSCs isolation, ex vivo culture and characterization

According to the policy approved by the local Ethical Committee (S. Orsola-Malpighi University Hospital, Bologna, Italy), all tissue samples were obtained after informed written consent. FM-MSCs were isolated as described previously [48], [49]. Briefly, term placentas from healthy donor mothers obtained from caesarean sections were rapidly transferred to the laboratory, rinsed in Phosphate Buffered Saline (PBS) containing P/S (200 U/ml penicillin, 200 µg/ml streptomycin) and processed immediately. Amniotic membrane was separated from chorion through blunt dissection. Pieces of amniotic membrane were minced and subjected to 15-min digestion with 0.25% trypsin-EDTA solution. The supernatant (SN) was discarded and the tissue underwent a second digestion with 0.25% trypsin-EDTA solution, 10 U/ml DNAseI and 0.1% collagenase IV solution in DMEM (all from Sigma-Aldrich, St. Louis, USA). Larger pieces of tissue were allowed to settle under gravity for 5 min at 37°C, while each SN was transferred to a fresh tube, neutralized with Fetal Bovine Serum (FBS, Biochrom, Berlin, Germany), then spun down at 600×g for 10 min. Each pellet was resuspended in 5 ml of DMEM containing 20% FBS, and P/S. Cells were seeded in 25 cm^2^ flasks and grown at 37°C in 5% CO_2_. Non-adherent cells were removed after one week and the medium (with 10% of FBS) was subsequently changed every four days. When the culture reached 90% confluence, cells were passaged using 0.25% Trypsin-EDTA.

Surface antigens were characterized as described elsewhere [50]. Briefly, trypsinized cells were resuspended in cell culture medium (10% FBS), incubated on ice for 10 min, washed with Dulbecco's phosphate-buffered saline (PBS, pH 7.2, 10% FBS, Invitrogen, Carlsbad, USA), and labeled with 1 µg/10^6^ cells FITC-conjugated antibodies for 40 min at 4°C in the dark. Cells at the 5^th^ passage number were used for all infection experiments. Sometimes cells at later passage numbers (up to the 20th) were used, without any appreciable difference in infectability and virus yield.

### Infection

Target cells were seeded either on 24- or 6-well culture plates at a cell density of 10^4^ cells/cm^2^. When required, cells were layered onto coverslips in 24-well plates, grown until they reached approximately 80% confluence and infected as described below.

### Viruses

An HSV-1 clinical isolate at low passage from our diagnostic laboratory was used in most experiments. The HSV-1 derived replication deficient viral vector TOZGFP-HSV1, carrying deletions in the immediate early genes ICP4, ICP22 and ICP27, and mediating expression of GFP under the control of the ICP22 promoter was also utilized, as indicated [51]. The low passage HSV-2 (G) laboratory strain was used in this study. Virus stocks were prepared and titrated in Vero (HSV-1) and BHK (HSV-2) cells. Briefly, SNs from infected cells approaching 100% cytopathic effect (cpe) were collected and subjected to three rounds of 20 sec sonication with a Bandelin Sonoplus sonicator, Model HD-2200 with an SH213G probe (Bandelin Electronic GmbH, Berlin, Germany) allowing a 1 min cooling-off period between each sonication. Cellular debris were pelleted by centrifugation at 800×g for 20 min at 4°C, and the SN were aliquoted and stored at −70°C. Titration was performed by thawing frozen aliquots, which were sequentially tenfold diluted and used in triplicate to infect Vero or BHK cells layered onto coverslips in 24-well plates for 1 h at 37°C, in a 5% CO_2_ atmosphere. The inoculum was then removed, cells were rinsed with 1 ml PBS and fed with fresh medium. 6 h post-infection (pi) a neutralizing antibody was added to the medium in order to avoid extracellular spread of progeny virions. Plaques were counted three days pi in wells where the dilution of the viral stock allowed less than 10 plaques to appear, and the correspondent titer was calculated. FM-MSCs were infected with virus stocks at a multiplicity of infection (moi) varying from 1–2.5 plaque forming units (pfu)/cell. Infection was performed for 1 h at 37°C in a humidified CO_2_ incubator, then the inoculum was removed and fresh medium added.

For VZV, a clinical isolate at low passage from our diagnostic laboratory was used in all experiments. Virus stocks were prepared in Vero cells by infection with cell-associated virus at a ratio 1∶4, infected to uninfected cells in 75 cm^2^ plastic flasks, harvesting infected cells when cpe was >80% with a cell scraper in their medium. Cell debris were subjected to three rounds of sonication as for HSV, except that cell debris were not separated from the medium after sonication, but were directly aliquoted and stored at −70°C. Rough titrations were performed by infection of Vero cells with serial dilutions of the frozen stocks in 24-well plates, overlaying a 0.6% agarose containing culture medium 24 h pi and counting plaques at three days pi FM-MSCs were infected with VZV virus stocks at a multiplicity of infection (moi)<1 pfu/cell. Infection was performed for 1 h at 37°C in a humidified CO_2_ incubator, then fresh medium was substituted to the inoculum. Sometimes HEL cells were used for titration of virus yielded by FM-MSCs or HEL infected monolayers, following the procedure described above.

As far as HCMV is concerned, we used the AD169 laboratory strain and the low passage endotheliotropic TB40 strain. Viral stocks of TB40 were prepared in HUVECs, while AD169 was prepared in HEL. Viral stocks were titrated in HEL as described elsewhere [50]. FM-MSCs and HEL were infected with HCMV viral stocks with a moi varying between 0.1 to 2.5 pfu/cell. Infection was performed for 1 h at 37°C in a humidified CO_2_ incubator, then fresh medium was substituted to the inoculum.

HHV-6 viral stocks were obtained as previously described [52]; HHV-6 variant A (strain U1102) was grown in the T lymphoid J-Jhan cell line [53]; HHV-6 variant B (strain CV) was grown in the Sup T1 cell line [54]. A single inoculum was prepared and used for all infection experiments to avoid variability in the efficiency of infection due to differences in inocula. Briefly, cell-free viral inocula were obtained by pelleting a total of 500 ml of cell cultures (at a concentration of 1×10^6^ cells/ml) infected with HHV-6A or HHV-6B exhibiting complete cpe. The cells were lysed by 3 cycles of rapid freezing and thawing followed by sonication (3 cycles of 5 sec at medium power with 10 sec intervals in water bath sonicator). Cleared cellular content was added to culture supernatant and virions were collected by centrifugation at 20000×g at 4°C. Virus particles were purified by density centrifugation using Optiprep self-forming gradients (Sentinel, Milan, Italy) at 5.8×10^4^×g for 3.5 h at 4°C. Collected virions were suspended in PBS containing 1% bovine serum albumin, fractioned in aliquots of 100 µl and stored at −80°C until use. Prior to use, virus stocks were treated with DNase-I and RNase A, to eliminate free viral nucleic acids eventually present in the preparation. HHV-6 viral stocks were quantitated by real time PCR as already described [55] using primers which amplify both HHV-6 variants. The standard curve was generated by amplification of a plasmid containing the targeted HHV-6 sequences. The method had a 6-log dynamic range and a sensitivity of 20 copies/ml. FM-MSCs were infected with HHV-6A or B with a moi of 50 genomes/cell. Infection was performed for 2 h at 37°C in a humidified CO_2_ incubator, then the inoculum was removed and fresh medium added.

HHV-7 Strain CZ [56] was grown in the Sup T1 cell line. Cell free viral inoculum was obtained as previously described [54]. Briefly, 1 l of HHV-7 infected cell cultures exhibiting complete cpe was centrifuged at 900×g, resuspended in 2 ml of FCS supplemented with RNase (50 µg/ml; Boehringer Ingelheim, Milan, Italy) and disrupted by four cycles of freezing in liquid nitrogen and thawing at 37°C. The resulting inoculum was completely free of living cells, as checked by microscopic observation and cultivation, and was also analyzed by reverse transcription PCR (both for β-actin and for a panel of viral mRNAs) to ensure that RNA was completely absent. 1×10^6^ FM-MSCs were infected with 400 µl of the resulting inoculum. Infection was performed for 2 h at 37°C in a humidified CO_2_ incubator, then the inoculum was removed and fresh medium added.

The B95-8 cell line was used to generate infectious EBV virus. Cells were propagated in complete RPMI medium at 37°C and an atmosphere of 5% CO_2_, and fed 2 times per week, maintaining cell density between 0.5–1.0×10^6^/ml. On day 0, 12-O-tetradecanoylphorbol-13-acetate (TPA) was added to a final concentration of 25 ng/ml together with sodium butyrate to a final concentration of 4 mM. The cells were harvested on day 5. Cells that were not induced were also harvested on the fifth day. SN from TPA induced B95-8 cells was used to infect FM-MSCs after clearance at low speed of cellular debris and subsequent filtration through a 0.45 µm-size-pore filter to remove any remaining debris. The same SN was ultracentrifuged at 150,000×g for 90 min in a Beckman SW55 rotor and the resulting SN used to perform mock infections. Titration of inocula was performed by determining the number of genomes present in serial dilutions of the final suspension with a real time PCR, targeting the exon 4/5 of the terminal protein gene with primers EB-1 AACATTGGCAGCAGGTAAGC and EB-2 ACTTACCAAGTGTCCATAGGAGC, and producing a 182 pb amplicon [57].

FM-MSCs infection was performed with a moi of 100 genomes/cell at 37°C in a humidified CO_2_ incubator for 2 h, then the inoculum was removed and replaced with fresh medium. Mock infections were performed in the same conditions. Primary human B lymphocytes were infected or mock infected in the same conditions in parallel.

BC-3 cells, a KSHV/HHV-8 positive primary effusion lymphoma (PEL) – derived cell line, were used as a source of HHV-8 and virus was purified by a modification of the protocol described by Caselli and co-workers [58]. Cells were propagated in RPMI supplemented with 20% fetal bovine serum at 37°C and an atmosphere of 5% CO_2_, fed every 2–3 days for 3–4 weeks until a minimal amount of 2×10^8^ cells was obtained. Cells were then treated with TPA at 20 ng/ml (Sigma-Aldrich, St. Louis, USA) for 60 h, subsequently centrifuged and the supernatant was collected. The cell pellet was lysed by three freeze-thaw cycles, centrifuged to eliminate cell debris, pooled with the supernatant, filtered through a 0.45-µm-pore-size filter, and concentrated at 4°C overnight with 7% polyethylene glycol 8000 (Sigma-Aldrich, St. Louis, USA). The suspension was centrifuged at 1.5×10^4^×g for 2 h at 4°C and the pellet was resuspended in a small amount of phosphate-buffer saline (PBS) and purified through a 25% sucrose cushion (2.65×10^4^×g for 3 h in a Beckman JSW-13-1 rotor). The pellet was resuspended in PBS and layered onto a 40–70% sucrose gradient and centrifuged at 2.65×10^4^×g for 1 h at 4°C in a Beckman JSW-13-1 rotor. The visible band was collected, dialyzed O/N against PBS, aliquoted and then stored at −80°C. Serial dilutions of this stock suspension were titrated by determining the number of genomes, with a real time PCR targeting the ORF73 gene, using primers HH8-1 5′TTGGGAAAGGATGGAAGACG and HH8-2 5′AGTCCCCAGGACCTTGGTTT, which determine an amplification product of 346 bp [59]. Infection of FM-MSCs and HUVECs was performed with a moi of 50 genomes/cell, at 37°C in a humidified CO_2_ incubator for 2 h. After that the inoculum was removed and replaced with fresh medium.

### Nucleic Acid Purification

At specific time-points pi cells were harvested by trypsinization, washed twice in cold sterile PBS, collected by centrifugation at 700×g and stored at −80°C until processed. For nucleic acid extraction and purification cells were thawed, resuspended in 100 µl of cold PBS and total nucleic acid were extracted with the NucliSENS easyMAG automatic extractor (bioMérieux sa, Marcy l'Etoile, France), following the manufacturer's instructions. Purified material was used both for DNA and RNA analysis.

### Polymerase chain reaction (PCR) for detection of viral DNA in donor FM-MSCs

2 multiplex PCR assays were performed, HERP-1 for HSV-1 and 2, VZV, EBV and HERP-2 for HCMV, HHV-6, HHV-7 and HHV-8. Primers included were previously described by others [57], [59], [60].

### PCR analysis for detection of HHV-6A and B, and HHV-7 in infected FM-MSCs

To determine which samples harbored viral genomic DNA sequences, 1 µg of DNA (corresponding to 1.5×10^5^ cells) was analyzed by nested PCR by using primers amplifying the U42 gene both for HHV-6A and B, and HHV-7. All primers were derived from the published HHV-6 and 7 sequences [61], [62]. The primer sequences as well as the PCR conditions were as previously described [53], [54], [63], [64].

### Reverse Transcription-PCR (RT-PCR) for the detection of viral transcripts in infected FM-MSC

DNA enzymatic degradation was performed by means of DNAse I Amplification Grade (Invitrogen, Calrsbad, CA, USA). RT-PCR was performed with the SuperScript™ III CellsDirect cDNA Synthesis System (Invitrogen, Calrsbad, CA,USA), and specific sequences were PCR amplified in an automated thermal cycler, using the Perfect Taq Plus amplification kit (5 PRIME GmbH, Hamburg, Deutschland) according to manufacturer's instructions. For detection of EBV, the three splicing form of EBNA-1 transcript were amplified using the set of oligonucleotides previously published by Chen and co-workers [65], following their working condition. BZLF1 first round RT-PCR was performed using 1 µl of cDNA in 50 µl reaction volume and 1.5 mM MgCl_2_ final concentration, using oligonucleotides BZLF1-OUT-Fw 5′AGCAGACATTGGTGTTCCAC and BZLF1-OUT-Rv 5′ACATCTGCTTCAACAGGAGG. Amplification reactions were carried out incubating samples at 95°C for 2 min then a 35 round cycle constituted by 95°C for 30 sec, 55°C for 30 sec and 72°C for 30 sec incubations, followed by a final incubation at 72°C for 10 min. BZLF1 nested RT-PCR was performed with the same amplification condition in combination with primers BZLF1-IN-Fw 5′ACGCACGGAAACCACAAC and BZLF1-IN-Rv 5′GCGCAGCCTGTCATTTTCAG. RT-PCR for the detection of HHV-6A and HHV-6B transcripts was performed as described elsewhere [53], [66], while HHV-7 specific transcripts were analyzed as described in [54]. In the case of HHV-8, ORF73 transcript was amplified using oligonucleotides ORF73-OUT-Fw 5′GAAGTGGATTACCCTGTTGTTAGC and ORF73-OUT-Rv 5′AGTCCCCAGGACCTTGGTTT and 2.5 µl of cDNA as template. ORF 73 semi-nested PCR was performed using oligos ORF73-OUT-Fw and ORF73-IN-Rv 5′TATCTCAGGCCTTCCAGTTT ORF50 transcript was amplified with oligonucleotides ORF50-OUT-Fw 5′GCCCTCTGCCTTTTGGTT and ORF50-OUT-Rv 5′GATGATGCTGACGGTGTG. Semi-nested PCR was performed with oligonucleotides ORF50-IN-Fw 5′ GCAAGGTCACTGGACTGTC and ORF50-OUT-Rv. All amplification reactions were carried out in a 50 µl reaction volume, with 1.5 mM MgCl_2_ final concentration, using 2.5 µl of DNA as template. A common amplification protocol was used, with the following conditions: 95°C for 2 min, 40× (95°C for 30 sec, 60°C for 30 sec and 72°C for 60 sec), 72°C for 10 min final amplification.

### Fluorescence Microscopy

Cells were processed for IIF analysis essentially as described previously [67]. Following fixation with paraformaldehyde 4% (w/v) and subsequent membrane permeation with 0.1% TritonX-100 treatment, cells were incubated with primary antibody for one h at 37°C in a humidified chamber. Three wash steps were subsequently performed in PBS, five min each at room temperature (RT). A secondary antibody, labeled with a fluorophore and raised against the species producing the primary antibody, was then incubated with the cells, in the same conditions as for the primary antibody. Antibody dilutions varied and were generally in compliance with manufacturers instructions. When human serum samples from our serotheque collection were used, dilution was generally 1∶50. Cells were then washed for five min three times in PBS, and subsequently fixed with paraformaldehyde 4% (w/v). Sometimes nuclei were stained by incubating with DAPI (1 µg/mL) for 4 min at RT. Samples were washed with PBS, mounted on coverslips in PBS/glycerol 50% (v/v), and imaged using a Nikon Eclipse E600 microscope equipped with a Nikon DXM1200 digital camera and a Nikon Plan Fluor 40× objective (Nikon, Tokyo, Japan), essentially as previously [68]. Live cells grown on Willcodishes (Willcowells, Amsterdam, The Netherlands) were infected with TOZGFP-HSV1 [51] and analyzed for GFP expression using a Nikon Eclipse TE2000-U inverted microscope equipped with a Nikon DXM1200 digital camera and a Nikon Plan Fluor 40× objective (Nikon, Tokio, Japan), as previously [69]–[71]. Mock infected cells were always processed in the same conditions and observed as a control.

## Results

### Herpesvirus genomes are not found in term placenta derived MSCs from healthy (seropositive) individuals

FM-MSCs obtained from term placentas of 6 donors, were analyzed for the presence of herpesvirus genomes by means of 3 multiplex PCR assays developed in house, HERP-1 for HSV-1 and 2, and VZV, HERP-2 for HCMV, HHV-6, and HHV-7 and HERP-3 for EBV and HHV-8 [72]. No traces of any herpesvirus genome were found in any of the six populations of FM-MSCs used in this study (data not shown).

### Permissivity of FM-MSCs to herpesviruses

To assess the infection efficiency of viral stocks, cell lines permissive to HHVs infection were infected with the same moi used for FM-MSCs and time-points were collected.

All RNA samples were analyzed by PCR without retrotranscription (RT) to check the total absence of DNA contamination. No contaminant DNA was present in any sample (see figures, RT-). Furthermore the efficiency of RT was monitored by amplification of the human β-actin cDNA, after a 1∶100 dilution.

### Alpha herpesviruses

#### HSV-1 and HSV-2

**Figure 1:**
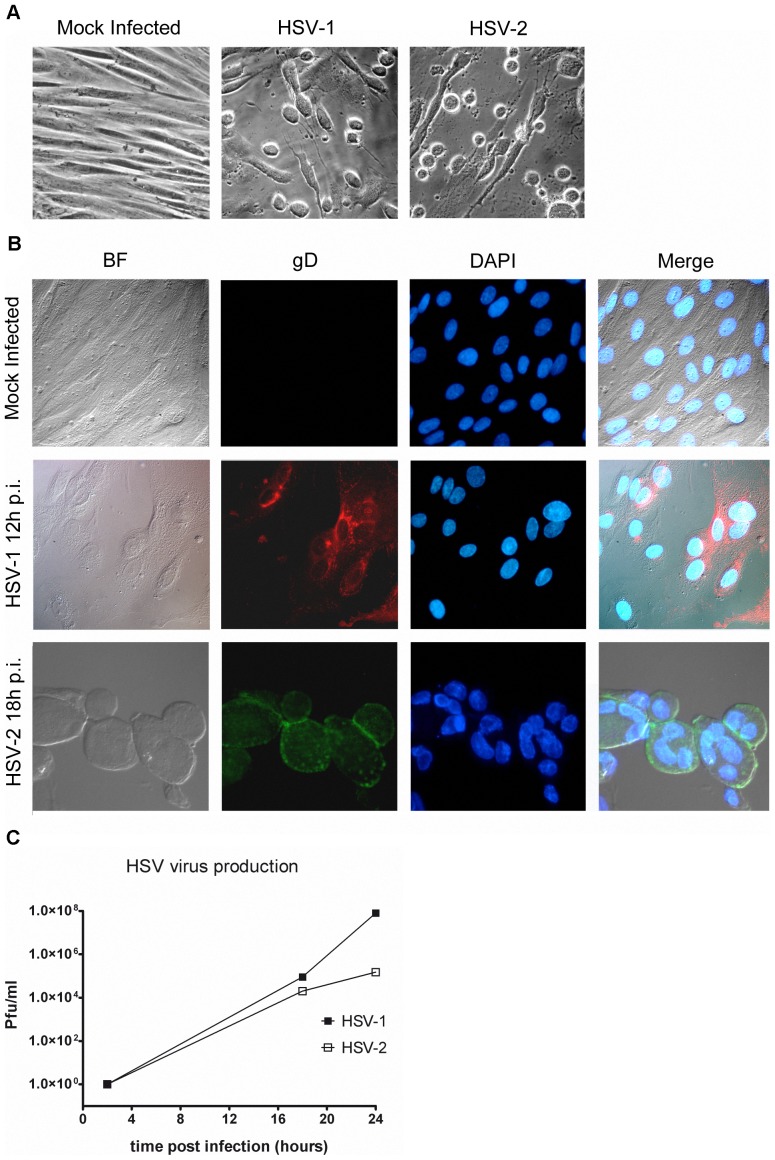
FM-MSCs are susceptible to HSV-1 and HSV-2 infection. (A) FM-MSCs were either mock infected (*left panel*) or infected with >1 pfu/cell of the indicated viruses before being analyzed with an inverted optical microscope as described in the Materials and Methods section. The typical cytopathic effect shown is observed starting 12–18 h pi; (B) FM-MSCs were either mock infected (*top panels*) or infected with >1 pfu/cell of the indicated viruses before being fixed and processed for IFF using a mAb directed against the late glycoprotein gD to detect infected cells, and DAPI to visualize cell nuclei. The bright field (BF) is shown in the *left panels*, and a merged image of all channels is shown in the *right panels*. Formation of typical syncytia in infected cells is evident starting at 18 h pi, as shown in the HSV-2 *bottom panels*; (C) SNs of infected FM-MSCs were collected at the indicated times pi and used to reinfect Vero (for HSV-1) or BHK (for HSV-2) cells to quantify infectious virion yields in the extracellular fluid of FM-MSCs by means of plaque assays as described in the Materials and Methods section.

MSCs originated from bone marrow have been shown to be permissive to HSV-1 [43], while heparan sulfate has been suggested as a major determinant for virus entry in adipose tissue derived MSC [73]. To investigate the degree of permissivity of FM-MSCs to HSV-1 and HSV-2, monolayers of FM-MSCs were infected with a clinical isolate of HSV-1, or the HSV-2 laboratory strain G. When infected cells were observed under an optical inverted microscope, the typical HSV-induced cpe became apparent from 16–24 h pi at high moi (1A), whereas cpe was observable only at later times after infection at low moi. Furthermore, IIF analysis allowed the specific detection of the L glycoprotein gD (Fig. 1B). Observation of cells infected at high moi from 18 h pi allowed the identification of polykaryons (Fig. 1B, bottom panels), whose formation requires L viral proteins gB, gD, gH and gL, that can be synthesized only after IE and E transcripts have been translated and DNA replication has been initiated. To verify that infectious progeny virus was released in the SN of infected cells, SNs were collected from infected cells at 2, 18 and 24 h pi and infectious virus was titrated on Vero or BHK cells in the case of HSV-1 or HSV-2, respectively. Both low and high moi infections proceeded until complete destruction of FM-MSCs monolayers. Our results show that infectious progeny virions are yielded from infected FM-MSCs, reaching titers of up to 10^7^ pfu/ml (Fig. 1C). In conclusion, FM-MSCs can be regarded as fully permissive for both HSV-1 and HSV-2 viruses.

Interestingly, when FM-MSCs and control Vero cells were infected with TOZGFP-HSV1, a replication defective viral vector developed for gene therapy applications by R. Manservigi and P. Marconi [51], the GFP transgene was readily detectable in both cell lines, starting from 20 h pi (Fig. S1).

#### VZV

**Figure 2:**
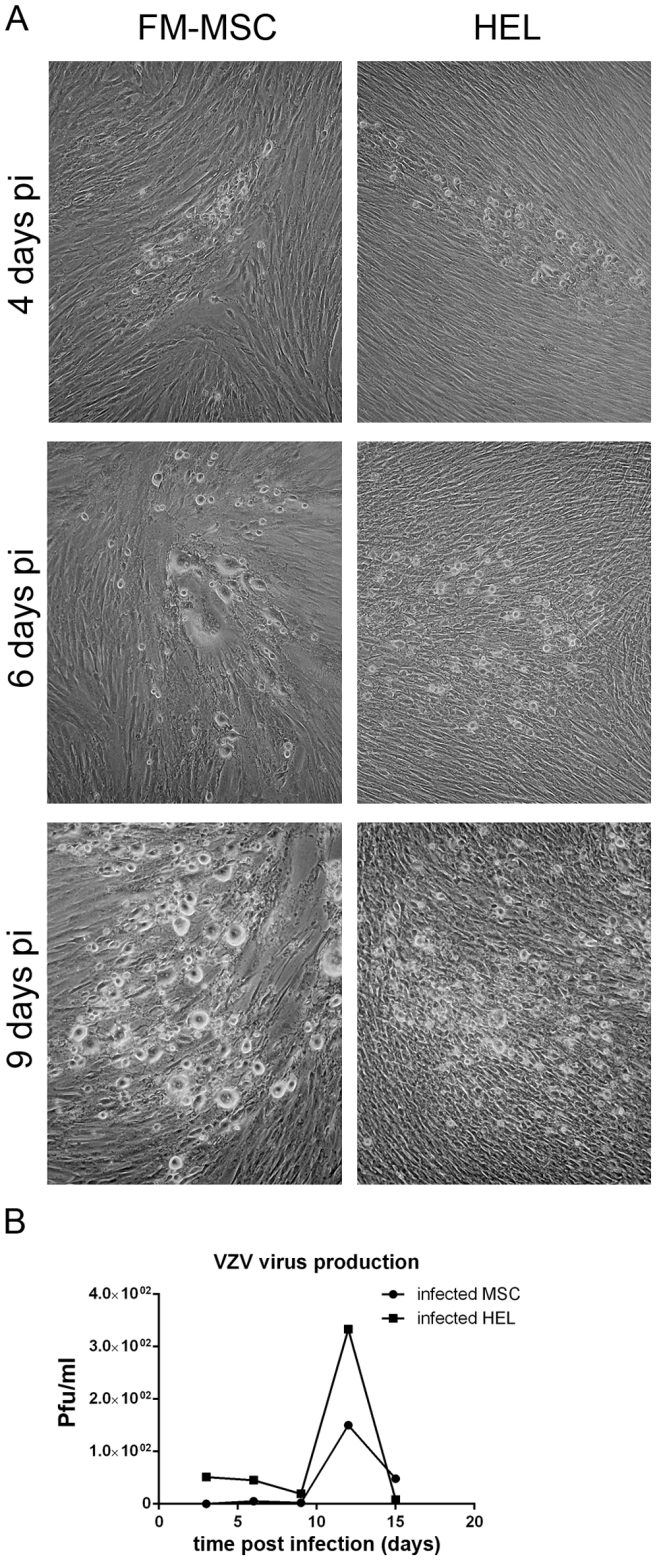
FM-MSCs are fully permissive to VZV infection. (A) HEL or FM-MSCs were infected with VZV at a moi<1 pfu/cell, as described in the Materials and Methods section. At the indicated time pi cells were analyzed by optical microscopy; (B) To determine if the cytopathic effect observed in (A) was due to productive VZV infection, infected cells were harvested at different time points pi along with their SNs, and processed as described in Materials and Methods to reinfect naive HEL cells. Infectious virus production was subsequently quantified by means of plaque counting. Each reported pfu value is referred to 10^5^ infected cells.

VZV genomic DNA has been PCR amplified from BM-MSCs isolated from two osteoarthritis patients in a sample of 18 patients [74]. However, susceptibility of MSC to VZV *in vitro* has never been addressed to date. We assayed the permissivity of FM-MSCs to VZV by infecting monolayers of FM-MSCs, using HEL as a positive control, with cell-associated VZV (moi<1). When infected cells were observed under an inverted optical microscope, foci of cytopathic effect became clearly visible from 4 days pi both in HEL and in FM-MSCs (Fig. 2A, left panels). At 6 days pi signs of cell fusion, a hallmark of VZV infection started to appear (Fig. 2A, middle panels). The effect was more pronounced in FM-MSCs, where larger polykaryocytes were observable as compared to HEL. This was even more evident at 9 days pi when cytopathic effect was generalized in both cell populations. The production of infectious progeny virions was assayed by titration of cell debris obtained by sonication of infected cells (moi<1 pfu/cell) collected at different time points pi, processed as described in Materials and Methods, and inoculated on HEL cells. As shown in Fig. 2B, infectious VZV was obtained from both infected HEL and FM-MSCs with a peak in the production of infectious progeny at 12 days pi. As shown in Fig. 2B, HEL cells yield 2–3 times more infectious progeny than FM-MSCs in our experimental conditions. Our results clearly show that FM-MSCs are fully permissive to VZV.

### Beta herpesviruses

#### HCMV

**Figure 3:**
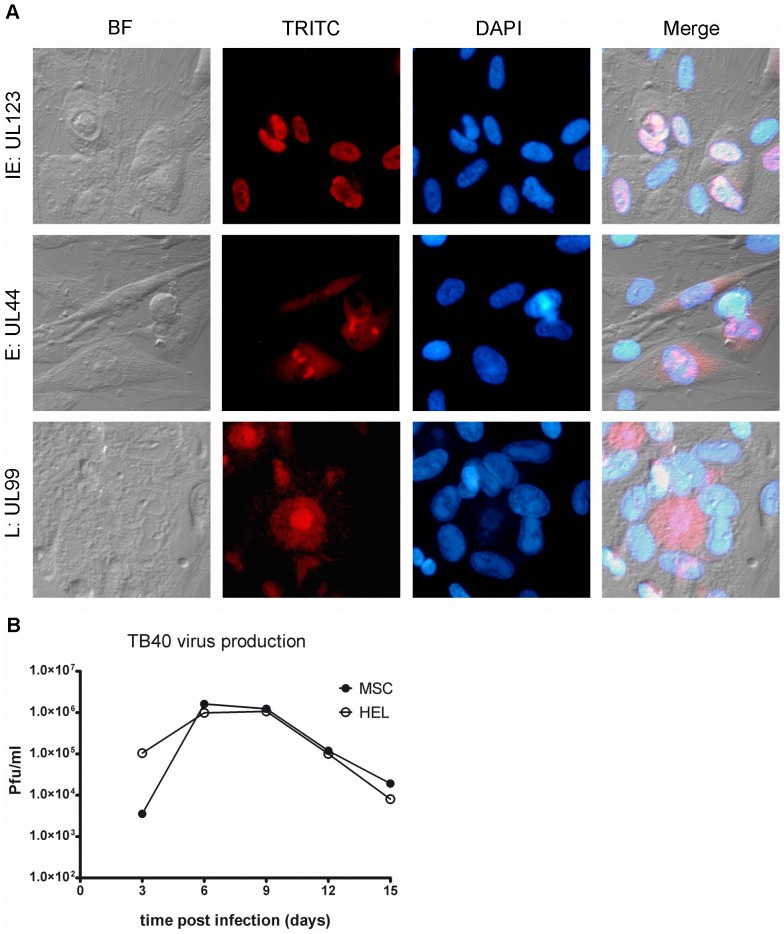
FM-MSCs are fully permissive to HCMV infection. (A) FM-MSCs cells were infected with HCMV (strain TB40) at a moi of 1 pfu/cell and fixed at 24 (*top panels*), 48 (*middle panels*) or 96 (*bottom panels*) h pi before being processed for IIF staining of HCMV temporal class representative antigens. Anti-IE (UL123), -E (UL44) or -L (UL99) specific antibodies were used, as indicated at the left of each row. The bright field (BF), the specific secondary antibody signal (TRITC) and the cell nuclei (DAPI) are shown, with merged images presented in the *right panels*; (B) SNs from HEL cells or from FM-MSCs infected in parallel were harvested at the indicated times pi and used as inocula to determine infectious progeny virion titers via plaque assays on HEL cells as described in the Materials and Methods section.

BM-MSCs have been shown to be susceptible to the AD169 strain [45] and to the clinical isolate TB40 [43] of HCMV. Here we similarly tested permissivity of FM-MSCs to both AD169 and TB40 viruses. To this end, we infected FM-MSCs, and HEL as positive controls, with both viruses at a moi of ≈1 pfu/cell. At this moi, a majority of cells in an infected HEL monolayer will allow progression of the viral replication cycle. The percentage of cells hosting a full viral replication cycle in a cell population with unknown permissivity to HCMV compared to that of a population of HEL infected in parallel with a comparable inoculum, reflects the permissivity of the cell type. Cells were fixed at different time points thereafter. IIF assays were then performed with antisera directed to specific virus encoded products. In order to determine if representative members of all temporal classes of viral genes were expressed in these cells, we analyzed the expression of IE, E, and L genes at 24–96 h pi in both cell populations. As shown in Fig. 3A, viral genes of all temporal classes of expression were expressed both in infected HEL and FM-MSCs. To analyze permissivity of FM-MSCs at the single cell level, counts were made of IIF positive cells for each antigen studied, at the indicated time points, and expressed as a percentage of the whole population. When the expression of the major IE gene UL123 was analyzed upon infection with TB40 from 24 to 96 h pi, 82% of the cell population was found positive at 24 h, 90% at 48 h, 93% at 72 h and 95% at 96 h pi. When the expression of the E gene ORFUL44 was analyzed at 24 h and 48 h pi, 58.5% and 72.6% of FM-MSCs stained positive respectively, while staining of the matrix protein expressed by the L gene ORFUL99 at 72 h and 96 h pi resulted in 87% and 96% positivity. When HEL cells were infected with TB40, or FM-MSCs and HEL were infected with a comparable inoculum of the AD169, we obtained similar results. As a whole these data suggest that FM-MSCs are fully permissive to HCMV, and that a replication cycle takes between 72 and 96 h to complete when cells are infected at a moi of 1 pfu/cell.

To evaluate the kinetics of replication of HCMV in FM-MSCs at high moi as compared to HEL cells, and verify that infectious progeny virions are released by infected FM-MSCs, we infected both cell populations at a moi of 10 pfu/cell with the TB40 strain. At this moi, the viral replication cycle is synchronized in all cells of the monolayer, and in HEL cells at 72 h pi progeny virions will appear in culture SNs. The medium of infected cell monolayers was therefore collected after 3 days, centrifuged to eliminate cell debris and frozen, while cells were fed with fresh medium. SNs were then collected in the same way with a 3 days time interval until the monolayers were completely lysed by the virus. A titration of all collected SNs was then performed on HEL. As shown in Fig. 3B virus yield in both cell populations reached a peak at 6 days pi and decreased gradually. HEL appeared more resistant to the virus than FM-MSCs, the latter monolayer being completely lysed 2–3 days before. This accounts for a 2 logs lower virus yield at 18 days pi in FM-MSCs compared to HEL cultures. Taken together these results indicate that FM-MSCs are fully permissive to HCMV.

#### HHV-6

**Figure 4:**
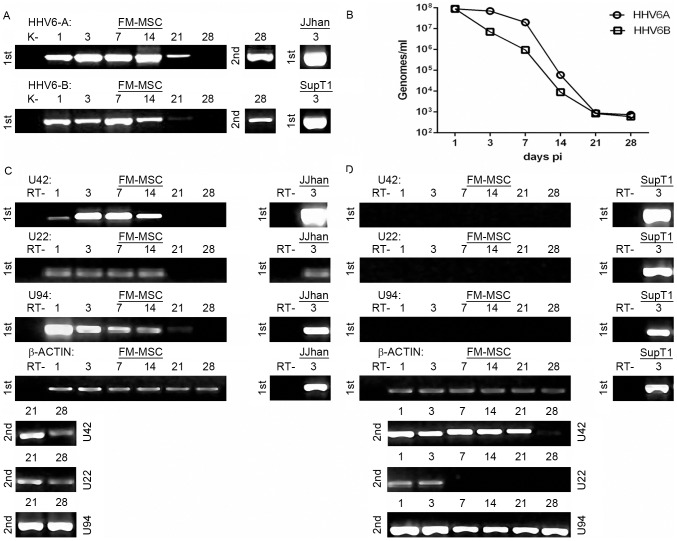
FM-MSCs are susceptible to HHV-6 A/B variants. FM-MSCs were infected with HHV-6 (variant A and variant B) at a moi of 50 genomes/cell and nucleic acids extracted at the indicated times pi. As positive control an equivalent number of cells permissive to HHV-6 productive infection (J-Jhan for HHV-6A and SupT1 for HHV-6B) were infected with the same viral stocks used for infecting the stem cells. A) Single step PCR (1st) and nested PCR (2nd) to detect HHV-6A and B genomes both in FM-MSC cells and J-Jhan and SupT1 cell lines. B) Real-time PCR quantification of HHV-6A and B genomes. C, D) single step (1st) and nested (2nd) RT-PCR for U42 (Early), U22 (Late) and U94 (latency associated) viral transcripts both in FM-MSC cells and J-Jhan and SupT1 cell lines infected with HHV-6A (C), or HHV-6B (D) variants. The β-actin RT-positive control is also shown. k- = mock infected FM-MSCs cells. RT- = PCR amplification of RNA samples without RT.

FM-MSCs were infected with a cell free inoculum of HHV-6A(U1102) or HHV-6B(CV). The inocula for the two viruses were standardized, quantitating viral genomes by real-time PCR. Infection was performed using moi of 50 viral genomes/cell. The cultures were analyzed at different times (1, 3, 7, 14, 21 and 28 days pi). Infected cultures had a morphology similar to uninfected cultures and no cpe was detected (data not shown). DNA was extracted at all time points and analyzed by single round PCR and nested PCR for the presence of HHV-6A and HHV-6B DNA, with a PCR specific for U42. For both variants, viral DNA was present at all time points, up to 21 days pi after single step PCR, and up to 28 days pi by nested PCR (Fig. 4A). These results suggested that the amount of intracellular viral DNA decreased over time, and therefore real-time quantitation of viral DNA was carried out. The results show that the amount of intracellular HHV-6 DNA decreased from approximately 5×10^7^ genome equivalents in 1.5×10^4^ cells 24 h pi to 5×10^2^ at 28 days pi, and that both variants exhibited the same behavior (Fig. 4B). Therefore, it is plausible to postulate that viral DNA persisted in infected cells with low levels of viral replication.

To determine the replicative state of HHV-6 in infected FM-MSCs, viral transcription was analyzed by RT-PCR for the presence IE (U42) and L (U22) viral lytic transcripts. The analysis included also U94/*rep*, a latency-associated transcript that is expressed at low levels also during productive infection [55]. The sensitivity of PCR reactions was similar for all genes, detecting consistently 10^3^ target molecules after first round PCR and 10–50 target molecules after nested PCR. Cells infected with HHV-6A showed the presence of U42 and U22 transcripts by first round PCR until 14 days pi, and the transcript of U94 was detected till 21 days pi. All three transcripts persisted as long as 28 days pi, as shown by nested PCR (Fig. 4C). These results suggest that HHV-6A establishes a low level productive infection, characterized by scarce viral replication and decreasing amounts of viral products (both DNA and RNA) over time.

In the case of cells infected with HHV-6B, single round PCR failed to show the presence of viral transcripts, and nested PCR yielded positive results at all time points only for U42 and U94. The transcript of U22 was detected only during the first 3 days pi (Fig. 4D). These results suggest that viral replication compatible with lytic production took place only for a few days, and that afterwards HHV-6B infection was restricted to the early phase of the replication cycle, with persistence of IE transcripts (U42 and U94), and the absence of late mRNAs (U22).

#### HHV-7

**Figure 5:**
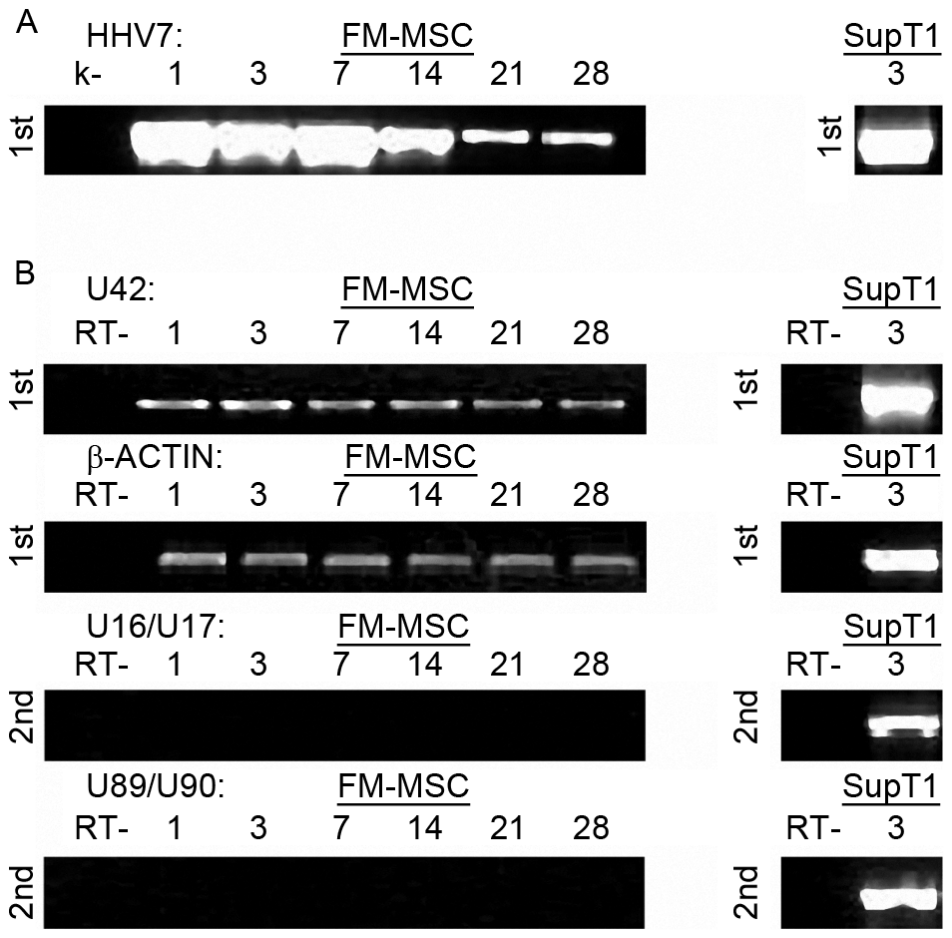
FM-MSCs are not permissive to HHV-7 productive infection. FM-MSCs (*left panels*) or SupT1 cells (*right panels*) were infected with HHV-7, and nucleic acids were extracted at the indicated times pi. A) HHV-7 genome single step DNA PCR amplification. B) Single step (1st) and nested (2nd) RT-PCR to detect HHV-7 transcripts. The β-actin RT-positive control is also shown. k- = mock infected FM-MSC cells. RT- = PCR amplification of RNA samples without RT.

FM-MSCs were infected with a cell free inoculum of HHV-7 strain CZ. The cultures were analyzed at different times (1, 3, 7, 14, 21 and 28 days pi). Infected cultures had a morphology similar to uninfected cultures and no cytopathic effect was detected (data not shown). DNA was extracted at all time points and analyzed by single round PCR, with primers and conditions specific for U42 of HHV-7. Viral DNA was detected at all time points, without need for nested amplification (Fig. 5A). No precise quantitation was performed, however the low intensity of the amplification bands at 21 and 28 days pi suggests that the amount of viral DNA decreased over time. Analysis of viral transcripts was carried out by RT-PCR, in order to monitor expression of U42, U16/17 and U89/90 mRNAs. All these genes belong to the IE transcriptional class and are expressed at high levels throughout viral replication, starting immediately after viral entry [54]. Only U42 mRNA was detected at all time points, with a faint band after single step PCR. U16/17 and U89/90 mRNA were not detected, even after nested PCR (Fig. 5B). On the whole, these results show that HHV-7 infects FM-MSCs cells, and that the virus persists without a full replication, expressing only some immediate-early functions [75].

### Gamma herpesviruses

#### EBV

**Figure 6:**
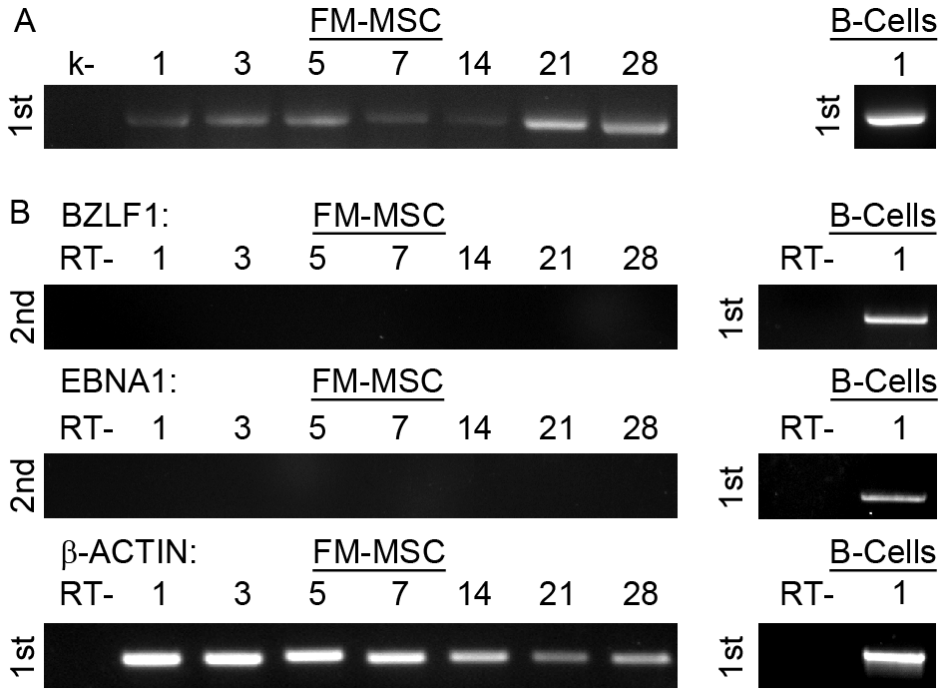
FM-MSCs are not permissive to EBV productive infection. FM-MSCs (*left panels*) or primary B lymphocytes (*right panels*) were infected with EBV, and nucleic acids were extracted at the indicated times pi. (A) Single step PCR (1st) targeting the EBV genome. (B) Single step (1st) or nested (2nd) RT-PCR targeting the indicated transcripts. k- = mock infected FM-MSC cells. RT- = PCR amplification of RNA samples without RT.

Since EBV infection depends on the receptor CD21, which is expressed on a limited number of cells, we decided to investigate whether CD21 is expressed on the surface of FM-MSCs by FACS analysis. Our results indicate that FM-MSCs express the EBV receptor (Fig. S2A), and that they are therefore likely infection targets. To assess the susceptibility of FM-MSCs to EBV infection, supernatant from a TPA induced B98-5 cell line was used to infect FM-MSCs and EBV susceptible human B lymphocytes at a genome/cell ratio of 100. The presence of the virus did not translate in any morphological change either at the cell monolayer or at the single cell level as observed by light microscopy. We set out to investigate if EBV was capable of penetrating inside FM-MSCs, and to this end we amplified DNA extraced from infected FM-MSCs and B-lymphocytes with EBV specific primers. As shown in Fig. 6A genomic DNA was amplified at up to 28 days pi, with generally very weak signals, while it was very strongly detected in genomic extracts of infected B-lymphocytes. Activation of viral transcriptional program was analyzed in genomic extracts of infected cells by RT-PCR (Fig. 6B). Our results confirm that EBV is capable of expressing both lytic (BZLF1) and latency associated (EBNA-1) transcripts during infection of B lymphocytes [76], [77]. By contrast, the same viral transcripts were undetectable in EBV infected FM-MSCs cell extracts by both single step and nested RT-PCR. These results taken together strongly suggest that EBV virions can enter FM-MSCs but transcription is not allowed while viral DNA persists for at least 4 weeks pi. Since the cell monolayer is confluent by day 3/4 pi, it is unlikely that failure to identify traces of viral transcripts or genome amplification is due to a dilution effect within a proliferating population of cells.

#### HHV-8

**Figure 7:**
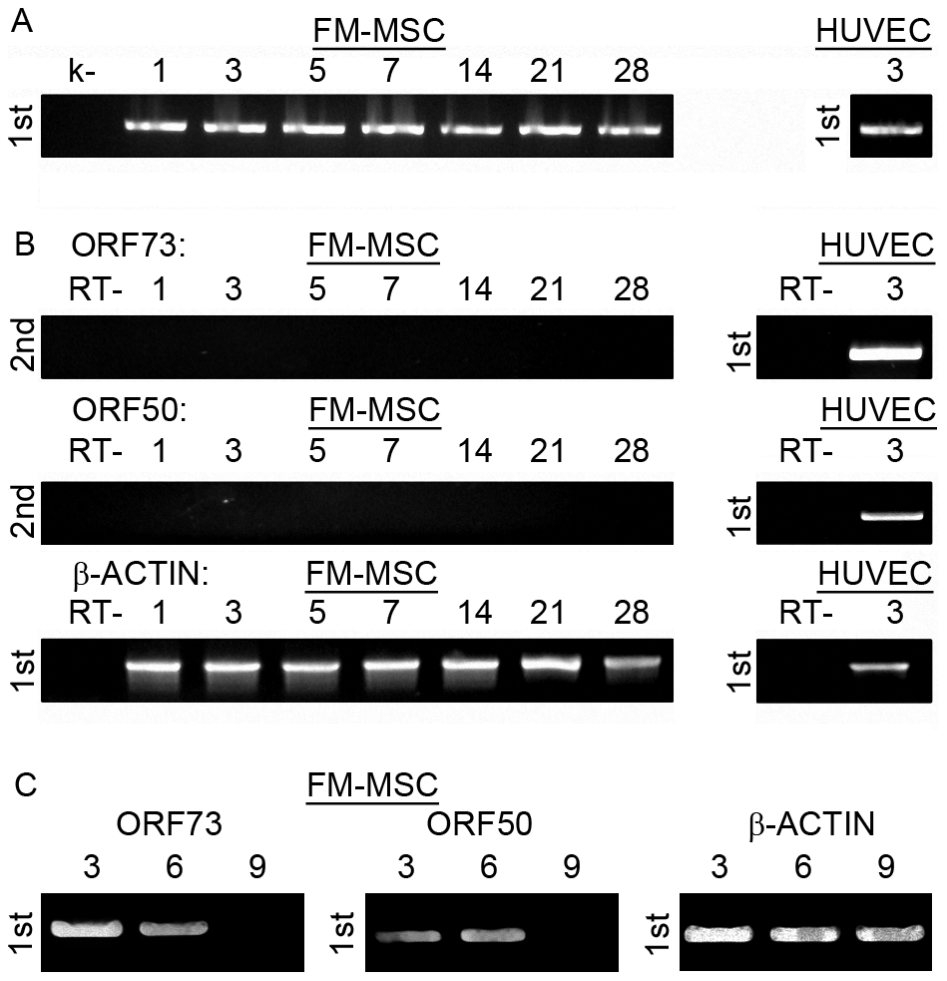
FM-MSCs are not permissive to HHV-8 productive infection. FM-MSCs (*left panels*) or HUVECs (*right panels*) were infected with HHV-8 at low (A, B) or high (C) moi, and nucleic acids were extracted at the indicated times pi. (A) Single step PCR (1st) targeting the HHV-8 genome. (B, C) Single step (1st) or nested (2nd) RT-PCR against the indicated transcripts. k- = mock infected FM-MSC cells. RT- = PCR amplification of RNA samples without RT.

It has previously been reported that HHV-8 predominantly infects CD14, CD34 and CD19-expressing cells [78]. Since CD14 and CD34 expression are not detected in FM-MSCs [48], we analyzed the surface of FM-MSCs membranes for the presence of CD19. Cytofluorimetric analysis showed consistent CD19 expression (Fig. S2B) suggesting that FM-MSC could represent a potential HHV-8 infection target. Furthermore MSCs from fetal BM can be infected latently *in vitro* by HHV-8, and virus persistence and expression of ORF73 has been documented for over 9 weeks in those cells [44]. To assess the ability of HHV-8 to infect FM-MSC, frozen stocks of purified virus were diluted in serum free medium and added to FM-MSCS at a moi of 50 genomes/cell. Serum free medium was used to establish mock-infected controls. HUVECs were infected in parallel as positive controls. Infected FM-MSCs remained unperturbed by the presence of the virus at all times pi as observed microscopically whilst infected HUVECs showed typical spindle like aspect, as previously described [58] (data not shown). The presence of intracellular and extracellular viral DNA was assessed by PCR amplification of nucleic acid extracts obtained at days 1 to 28, from cell monolayer extracts and extracellular fluid. Qualitative and quantitative analysis were performed on both extracts as described in Materials and Methods. Viral DNA was readily detected at all time points tested (Fig. 7A). Analysis of virus-encoded transcripts was performed on RNA extracted from mock infected and infected cells from 1 to 28 days pi, and analyzed by RT-PCR. Expression of ORF50 and ORF73 mRNAs, encoding for the major transactivator of HHV-8 lytic cycle and for the latency associated antigen, respectively, was analyzed by RT-PCR as described in the Materials and Methods section. As shown in Fig. 7B we did not detect any of the two transcripts at any time point analyzed from FM-MSCs, while they were both detectable after infection of HUVECs, accordingly with what previously reported (data not shown) [58]. When the infection was performed at high moi (>10^3^ genomes/cell) both ORFUL50 and ORFUL73 generated transcripts were detectable at 3 and 6 days pi, but not at later time points (Fig. 7C). Taken togheter this data are evocative of a general albeit short-lived activation of viral promoters in FM-MSCs, which is not followed by a lytic cascade of genomic transcription, nor by a detectable latency programme.

When quantitative analysis of virus genome copies content was implemented in high genome/cell ratio HHV-8 infected FM-MSC, 1 genome per 25 cells was detected at 3 days pi, 1 genome per 288 cells was found at 6 days pi, while at 9 days pi the amount of DNA was under the detection limit. Taken together these results suggest that HHV-8 is able to penetrate FM-MSCs and PCR detectable amounts of its genome persist for at least 4 weeks pi, however limited gene expression is allowed only following high moi and for less than one week.

## Discussion

The present work addresses for the first time the susceptibility of FM-MSCs to all members of the *Herpesviridae* family. We show here that FM-MSCs are susceptible to HSV-1, HSV-2, VZV and HCMV infection *in vitro*. While data presented in the present paper have been obtained using FM-MSCs at the 5th passage, preliminary data suggest that susceptibility remains unaltered from the 5^th^ to the 20^th^ passage, confirming the already described phenotypic stability of these cells throughout extensive expansion. Our results show that HHV-6A persists in FM-MSCs cells with low levels of complete viral expression, and therefore with a potential for production of infectious virus. On the other hand, HHV-6B and HHV-7 show a more restricted expression, with persistence of viral DNA and continuous transcription of some immediate-early genes. Even if late genes are not expressed, it is not possible to exclude the possibility that these viruses might undergo full replication under specific conditions of the host cell. HHV-8 persists for at least 4 weeks in infected FM-MSCs, while specific transcripts are detectable only upon high moi, but for not more than 6 days pi. Our data are consistent with the observation by Parsons and co-workers that expression of ORF73 is supported in fetal BM MSC upon infection at high genome/cell ratio [44]. In our experimental settings duration of ORF73 transcription in infected cells was shorter, very likely due to lower doses of inocula.

Ethical issues restrict the use of antenatal tissues as a source of SCs for scientific research and clinical applications. As an alternative one can rely upon the isolation of SCs from available fetal tissue samples, as with fetal bioptic or blood sampling and terminated pregnancies, or term placenta and postnatally retrieved FM, which yield mainly SCs with mesenchymal cell surface markers and morphology, MSCs. Amnion and chorion in particular constitute a valuable source of MSCs [11], [13], [14].

Herpesviruses are widely spread in the population, with seroprevalence rates ranging from a minimum of 30% for HCMV and 60% for HSV-1 in some Western developed Countries, to over 90% for VZV, HHV-6 and 7 and EBV in the rest of the world [79].

Our study, showing that FM-MSCs are fully permissive to members of the *Herpesviridae* family has implications in three medical research fields. In regenerative medicine in the first place, because herpesvirus infections remain a major challenge both in solid organ and hematopoietic stem cell transplantation, two settings in which the use of FM-MSCs is being implemented with growing expectations. In addition, it has been recently reported that HCMV infection of BM derived MSC hinders their differentiation abilities, suggesting that infection with certain herpesviruses could also affect FM-MSCs differentiation potential [34]. Awareness of the possible contamination of FM-MSCs isolated with viruses suggests the need for screening isolated cells for the presence of viral genomes, in order to avoid virus transmission to transplant recipients. Non-permissive infections in particular may act as transient viral reservoirs, allowing viral DNA to be carried on throughout manufacturing practices to the final cell product used in clinical applications. The possibility of reactivation events in response to stimuli originated pre- or post-infusion cannot be ruled out. Furthermore, the infection of transplanted tissues originated from the expansion of these cells could have adverse consequences on the outcome of the engraftment. While the legislation relative to cell products for therapeutic use is still under development, in Europe and in the United States, guidelines to good manufacturing practice for manufacturers of human cells have been issued. The two protocols “quality of Biotechnology products: viral safety evaluation of biotechnology products derived from cell lines of human or animal origin”, issued by the European Medicine Agency and “Reflection paper on stem cell-based medicinal products” issued by the European Committee for Advanced Therapies contain rather generic recommendations to check cells for contamination by not better specified human pathogenic viruses. The Tissue Guidance Document “Current Good Tissue Practice (CGTP) and Additional Requirements for Manufacturers of Human Cells, Tissues, and Cellular and Tissue-Based Products”, released by the U.S. Department of Health and Human services recommends testing cells of human origin for human viruses such as CMV, HIV-1 & 2, HTLV-1 & 2, EBV, HBV, and HCV. The document specifies that both master and working cell banks should be subjected to *in vitro* and *in vivo* viral testing. Finally, it states that selected species-specific testing for adventitious viruses should be performed at the different stages of manufacturing, including the virus cited above, and “other human viral agents”, as appropriate. Our work suggests that, in addition to the aforementioned investigations, donors of FM-MSCs may be considered for serologic screening for beta and gamma herpesviruses, and cells obtained from herpesvirus-seropositive donors tested for the presence of corresponding viruses at the time of banking and at the final step of the manufacturing process.

A second field of interest is that of vertical transmission of herpesviruses. Primary infection with HSV has been associated with spontaneous abortion, premature labor and intrauterine growth retardation, while primary VZV infection may result in stillbirth or cause congenital varicella, a syndrome associated with a significant mortality rate [80]. HCMV is the leading cause of congenital viral infection and the most common infectious agent of congenital malformations in developed countries. Viruses and other difficult to culture organisms have been postulated as the etiology of a number of obstetric and pediatric conditions of unknown cause, including stillbirth. Among herpesviruses, HCMV, HSV, and VZV may cause intrauterine deaths [81]. Infection of FM-MSCs with a member of the *Herpesviridae* family could have negative consequences on pregnancy and the fetus potentially at all times of gestation. MSC from vascularized FMs could be a first target for replication and dissemination to non-vascularized FMs and to fetal circulation, both serving as a reservoir of actively replicating virus, and interfering with the immune response of resident T cells. Since FM-MSCs have been shown to enhance fetal repair [82] and couple immunosuppressant abilities to antimicrobial effector function [83], their depletion by viral infection could further complicate viral damage to the fetus. Furthermore herpesviruses are known to cause chromosome breaks in infected cells [84], that could represent a teratogenic threat if considering that MSCs traffic between placenta membranes and the fetus [85], especially in very early developmental stages. Finally, since the placenta is not a barrier to maternal MSC trafficking, and BM derived MSC have been successfully infected *in vitro* with HSV-1 and HCMV, it is conceivable that vertical transmission could occur via this straightforward route. The severity of depletion or functional impairment of FM-MSCs during early gestation could depend upon maternal immune competence towards HCMV as occurs for cytotrophoblasts [86] and cause unsuccessful placenta implantation or abnormal placenta development, resulting in early failure. It is important to note here that our placenta-derived cells grow in cell culture conditions. The degree of their representativeness with respect to the original cell population constituting the stromal layer of mesenchymal tissue within fetal membranes has been considered with the intrinsic limitation due to an *in vitro* model. However, considering the high amount of MSC that can be isolated from a tiny area of fetal membranes, the number of passages needed to characterize the cell population is very limited (3–5), while our experiments were carried out at passage 5, when we would not expect major shifts in MSCs biochemical and biological characteristics would have occurred.

Finally our work is relevant to the field of virus mediated gene therapy applied to SCs. The importance of reprogramming FM-MSCs functions of interest or orienting their differentiation potential via gene transfer has been established and has a broad consensus [87], [88].

Because of their propensity to migrate to the sites of injury and the ability to expand rapidly, FM-MSCs have been considered as potential gene transfer vehicles to deliver therapeutic genes [6], [7], [89]. On the other hand, gene transfer to MSCs can be achieved by using viral vectors [90]–[92]. Our data, showing that infection of FM-MSCs with a replication deficient HSV-1 derived viral vector mediates GFP transgene expression (Fig. S1), strongly suggest that genetically modified versions of the herpesviruses may be used to this end. Using members of the *Herpesviridae* family for gene transfer can have several advantages: long-term transgene expression, repeat vector dosing with a minimal immune response as a result of the absence of helper viruses during viral packaging, and simultaneous delivery of large and multiple transgenes [93]. Hybrid HSV/EBV vectors have been used to enhance stability of transgene expression [94]. Different behavior in terms of genome stability and differential genome expression in infected cells may be advantageous to distinct goals as diverse as transient expression for the induction of differentiation or long term expression of therapeutic genes.

## Supporting Information

Figure S1 **FM-MSCs allow transgene expression following infection with an HSV-1 based viral vector.** FM-MSCs (*upper panels*) and Vero (*lower panels*) cells were infected with the replication defective viral vector TOZGFP-HSV1 (moi of c. 1), carrying deletions in three immediate early genes and the GFP cDNA inserted in the ICP22 locus. The bright field (BF) and the specific GFP signal (GFP) are shown, with merged images presented in the *right panels*.
(TIF)

Figure S2 **FM-MSCs express CD21 and CD19 cell surface proteins.** Flow cytometric analysis for the expression of surface markers CD21 (A) and CD19 (B) in FM-MSCs. Isotype control peak is shown in *black*.
(TIF)
