# Supplementary figures and images for: Susceptibility of Human Placenta Derived Mesenchymal Stromal/Stem Cells to Human Herpesviruses Infection
Source: PLoS One. 2013 Aug 5;8(8):e71412. doi: 10.1371/journal.pone.0071412 (PMC3734067; doi:10.1371/journal.pone.0071412)

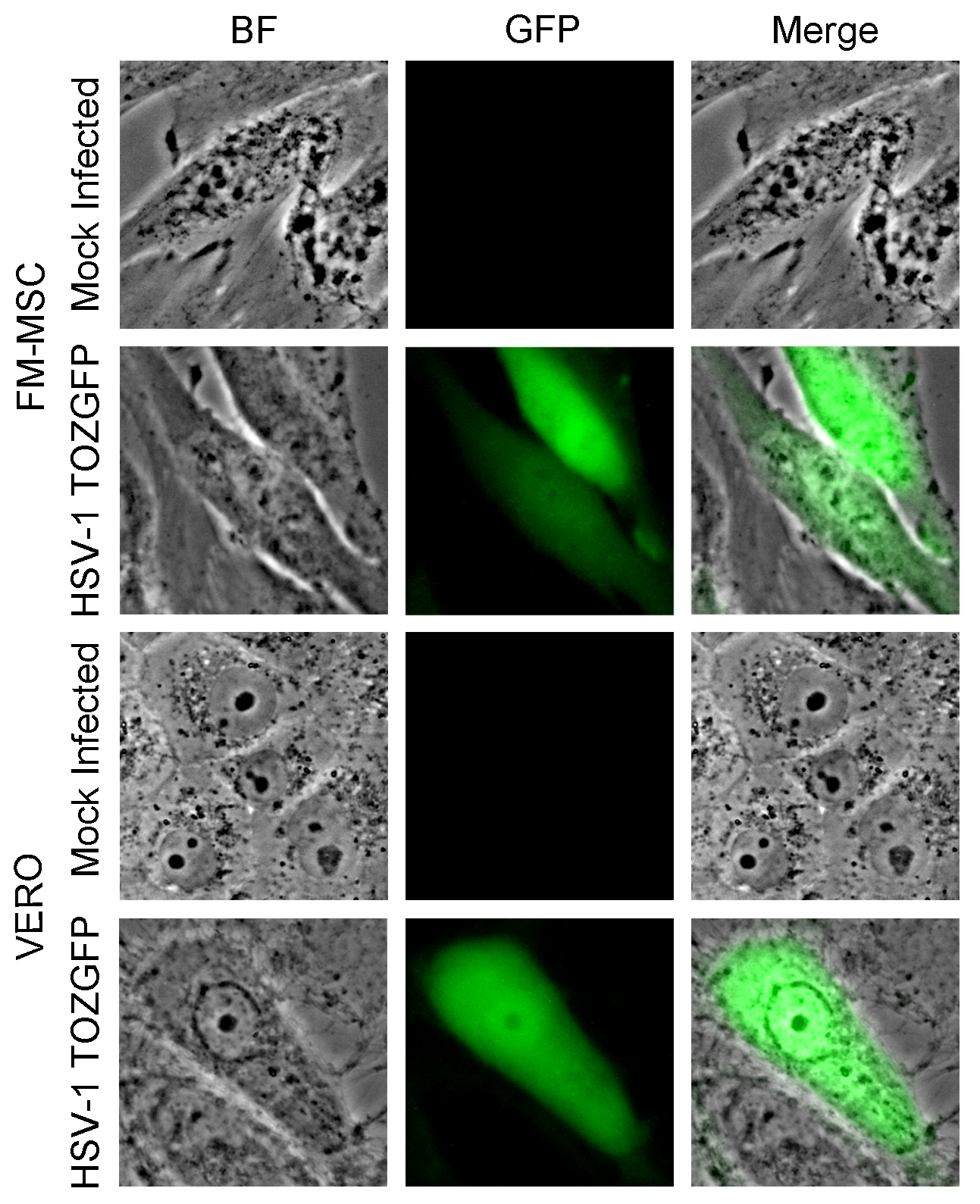

Supplement: Figure S1 — FM-MSCs allow transgene expression following infection with an HSV-1 based viral vector. FM-MSCs (upper panels) and Vero (lower panels) cells were infected with the replication defective viral vector TOZGFP-HSV1 (moi of c. 1), carrying deletions in three immediate early genes and the GFP cDNA inserted in the ICP22 locus. The bright field (BF) and the specific GFP signal (GFP) are shown, with merged images presented in the right panels. (TIF) [file pone.0071412.s001.tif]

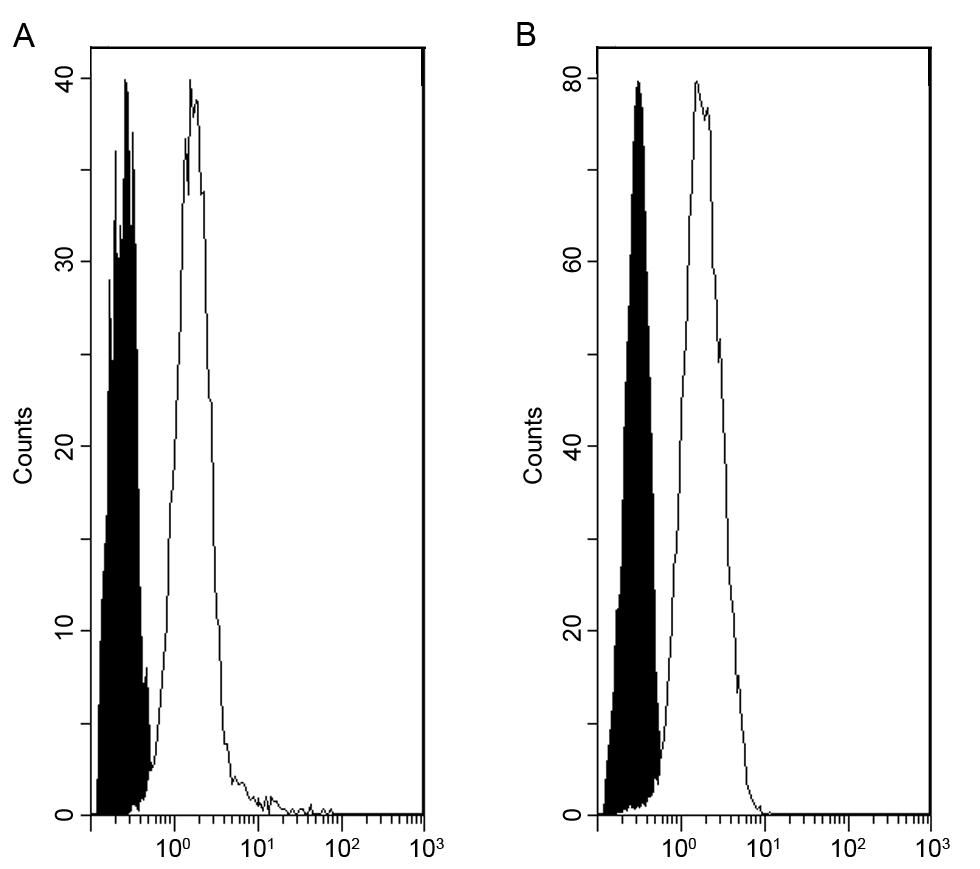

Supplement: Figure S2 — FM-MSCs express CD21 and CD19 cell surface proteins. Flow cytometric analysis for the expression of surface markers CD21 (A) and CD19 (B) in FM-MSCs. Isotype control peak is shown in black. (TIF) [file pone.0071412.s002.tif]
